# Magnetic dipole imaging of magnetite nanoparticles in brain tissue

**DOI:** 10.1039/d5ra08546b

**Published:** 2026-01-05

**Authors:** Leon Kaub, Stuart A. Gilder, Roger R. Fu, Barbara A. Maher, Gabriel Maxemin, Aaron T. Kuan, Andreas Büttner, Stefan Milz, Christoph Schmitz

**Affiliations:** a Department of Anatomy II, Faculty of Medicine, LMU Munich Pettenkoferstr. 11 80336 Munich Germany leon.kaub@lmu.de; b Department of Earth and Environmental Sciences, LMU Munich Theresienstr. 41 80333 Munich Germany; c Department of Earth and Planetary Sciences, Harvard University 20 Oxford St Cambridge MA 02138 USA; d Centre for Environmental Magnetism & Palaeomagnetism, Lancaster University Lancaster LA1 4YQ UK; e Department of Neuroscience, Yale School of Medicine 333 Cedar St New Haven CT 06510 USA; f Institute of Forensic Medicine, Rostock University Medical Center St.-Georg-Str. 108 18055 Rostock Germany

## Abstract

The human brain contains magnetic iron oxide nanoparticles in the form of magnetite (Fe_3_O_4_); however, the origin and physiological implications of these crystals remain debated. Due to their low concentrations in brain tissue (∼1–20 ng g^−1^), the identification and characterization of individual magnetic particles require nanometer-scale spatial resolution over large scan volumes. In contrast to conventional electron microscopy techniques that have field of views typically on micron scales, the Quantum Diamond Microscope (QDM), based on wide-field nitrogen-vacancy center imaging, can generate magnetic field maps over areas of several square millimeters while detecting nanoscale particles. Moreover, the QDM can directly quantify the strength and direction of the particles' magnetic moments. Operating the QDM in a high-sensitivity mode, coupled with long acquisition times, enabled the detection of magnetic moments as small as 3 × 10^−17^ Am^2^, corresponding to a magnetite particle diameter of approximately 50 nm, in maps covering 1.40 × 2.25 mm^2^. This is the highest magnetic moment sensitivity of wide-field magnetic microscopy >1 mm^2^ to date. In addition, collecting repeat, but slightly offset magnetic field maps resulted in the unique ability to distinguish sources within a sample from contamination and artifacts. By applying this technique to tissue, we demonstrate the detection of magnetic dipole-generating sources in human and rodent brain samples with the QDM. Detected particles span a size range of 60–135 nm, consistent with the larger end of magnetite particle sizes found by electron microscopy. These are the first direct magnetic observations of magnetite nanoparticles in brain tissue using quantum sensing techniques.

## Abstract 2

Quantum sensing with nitrogen-vacancy centers enabled the first direct magnetic observation and characterization of ferrimagnetic iron-oxide nanoparticles in brain tissue.

## Graphical abstract

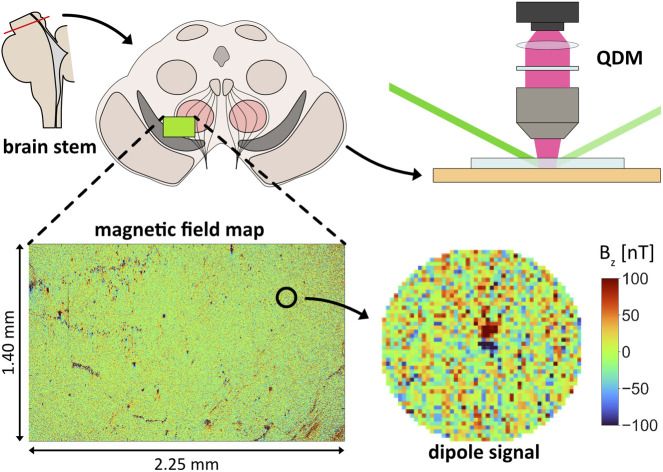

## Introduction

Several studies have documented the presence of magnetite (Fe_3_O_4_) nanoparticles in the human brain but their origin and physiological relevance remain debated.^1–5^ Early transmission electron microscopy (TEM) observations of euhedral magnetite crystals suggested endogenous biomineralization,^1^ a view supported by a systematic distribution of magnetite in the human brain.^3^ Others have proposed links to Alzheimer's disease,^6–8^ though this has been challenged by subsequent studies.^4,9^ Pollution-derived magnetite particles represent another potential source, with suggested entry pathways through the olfactory tract,^2^ the neuroenteric system,^10^ or circulation.^11^ Recently, magnetite concentrations in human brain stems were associated with liver disease.^5^

Most studies of brain magnetite have relied on bulk magnetic measurements, which do not reveal particle location. Yet *in situ* mapping of magnetite particles is essential to understand their physiological impact. TEM imaging provides nanometer-scale resolution; however, identifying single magnetite particles is extremely challenging given their small sizes and low concentrations in brain tissue (1–20 ng g^−1^).^3,4^ For example, based on the maximum magnetic moment observed previously^5^ (∼1 × 10^−6^ Am^2^ kg^−1^) and assuming a homogeneous distribution, the probability to find a 50 nm diameter magnetite particle in a 10 × 10 µm^2^ TEM image is ∼1 in 3000. Consequently, the identification of individual particles requires hundreds to thousands of TEM images,^12^ not to mention the potential for contamination during the extensive sample preparation process.^13^ Furthermore, crystallographic analyses of nanoparticles with TEM are hampered by beam-induced carbon buildup.^2^ As a result, only a few studies have imaged brain magnetite *in situ*,^2,10^ while most relied on extraction methods that destroy tissue architectures.^1,2,7,8,14,15^

The Quantum Diamond Microscope (QDM) provides an alternative for the detection of magnetic nanoparticles in tissue.^16^ By employing a thin (1–2 µm in our case) implanted layer of nitrogen-vacancy (NV) centers in diamond, it can generate magnetic field maps with a large (1.40 × 2.25 mm^2^) field of view (FOV) and high spatial resolution (1.17 µm). Although a spatial resolution in the micrometer range seems insufficient to image nanoparticles, mapping the magnetic field generated by such particles is feasible.^16,17^ Using the QDM facilitates non-destructive imaging with drastically reduced contamination risk. Furthermore, unlike electron microscopy, the QDM can directly quantify magnetic properties of each particle and allows identifying surficial contamination. Assuming a detection depth of 5 µm,^18^ the QDM can in principle detect hundreds of 50 nm-sized magnetite particles in brain tissue within a single FOV.

Wide-field NV magnetometry has been applied to biological systems, including magnetosomes in MTB,^19^ chiton teeth,^20^ malarial hemozoin crystals,^21^ and iron-rich organelles within pigeon cochleae.^22,23^ It has also been used to detect synthetic magnetite in cell cultures.^24,25^ By operating the QDM in high-sensitivity mode with long acquisition times, the detection of magnetic moments of ∼10^−17^ Am^2^, consistent with individual single-domain nanoparticles, is possible.^16^ Such resolution is essential to measure magnetic moments from individual magnetite nanoparticles.

Beyond biological samples, NV magnetometry is a versatile sensing technique with many applications for material sciences and chemical systems. At the highest sensitivity, it can detect paramagnetic ions^26^ and even nuclear spins,^27^ providing information on molecular aggregation, oxidation states, or magnetic ordering, which are relevant for studying heterogeneous catalysts or monitoring redox-active compounds.^28,29^ Generally, NV magnetometry can be used to probe a variety of nanoscale magnetic phenomena, complementing traditional imaging techniques. While the detection of single spins requires shallowly implanted, single NV centers,^29^ our approach using a large FOV with NV centers at ∼1 µm implantation depth allows screening large volumes with high sensitivity for detecting magnetic nanoparticles. Here, we apply QDM magnetic dipole imaging to human and rodent brain tissue, demonstrating that magnetic nanoparticles can be detected in brain tissue using quantum sensing techniques.

## Results and discussion

### Signal detection

**Fig. 1:**
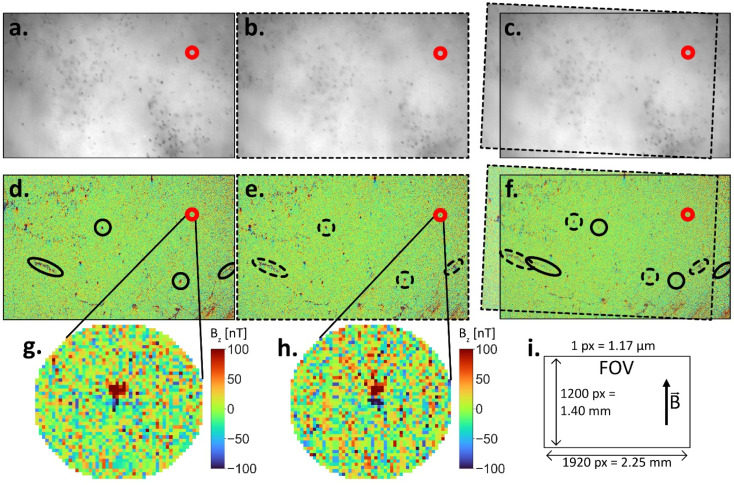
Repeat QDM map collection of the same FOV used to identify dipole signals from particles present in human tissue sample. (a and b) Reflected light image of sample 3985/2 (human mesencephalon) measured two times with a slight rotation between the two. Dark dots visible in both reflected light images, arising from neurons containing neuromelanin, serve as reference points to align the two images. (c) Both images superimposed after translating and rotating image 2 to match the FOV of image 1. (d and e) Corresponding magnetic field maps from images 1 and 2, respectively. (f) Superimposed magnetic field maps show that most signals translate after superposition (black ellipsoids mark some examples). These features stem from the diamond sensor and can therefore be considered artifacts. Only signals that remain at the same location in the superimposed magnetic field maps (red circles) originate from particles that reside in the sample (in this example only one source). (g) and (h) Show a magnification of the dipolar structure in the magnetic field map. (i) QDM FOV dimensions and sample magnetization direction 
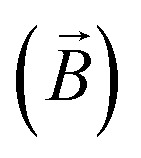
. Note that the dipoles detected in this example were well-aligned with 
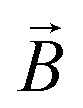
.

To image magnetic remanence carriers in brain tissue with the QDM, samples need to be in close contact with the diamond sensor of the instrument. We therefore embedded brain samples in methyl methacrylate or epoxy resin that allowed polishing of the sample's surface while preventing the sample from deforming during alignment. The distance between the sample and diamond sensor was kept to ≤1 µm, as confirmed by visible interference fringes between the sample surface and the diamond. We operated the QDM in a high-sensitivity mode, in which two resonant frequencies of ^15^N nitrogen-vacancy centers, split by ∼3 MHz due to hyperfine interactions, are driven simultaneously.^30,31^ This is achieved by microwave mixing, leads to an increase of contrast of fluorescence peaks, and lowers the root-mean-squared noise by a factor of ∼3.^32^ The high-sensitivity mode was combined with acquisition times between five and twelve hours per FOV (see Experimental section), leading to even higher signal-to-noise ratios. In this operating mode, a single QDM image resulted in a complex magnetic field map with multiple dipolar signals that originated from sources in the sample, artifacts from the diamond sensor, potential contamination, and noise. Distinguishing these sources based on single QDM maps is impossible, so we imaged each FOV twice, cleaning the diamond sensor and sample surfaces both times (Fig. 1).

This procedure allowed a direct identification of signals from unbound, surface contamination or noise if a potential source occurred in one map but not the other. In addition, the collection of duplicate maps enabled us to differentiate between signals originating from the sample and those from the diamond sensor. Signals from the diamond sensor arise from internal strain in the NV center layer and can thus be regarded as artifacts.^33^ The diamond, after cleaning, was reinstalled in the QDM with a slight shift compared to the first image. Similarly, the sample was reinstalled with a shift, typically of order 100 µm in an arbitrary direction. Sample alignment was achieved with bright-field LED reflected light images, which are taken using the same optical train as the magnetic field images and are therefore registered to the latter maps with pixel accuracy. After collecting both magnetic field maps, the reflected light images were used to determine the transform of the two images (translation and rotation) that was necessary to have both FOVs identically aligned in the sample frame of reference (Fig. 1a–c). The transform was then applied to the magnetic field maps (Fig. 1d–f). We consequently identified diamond artifacts by noting a shift from one field map to another (black ellipsoids in Fig. 1). Only signals occurring at the same position in both maps in the sample frame of reference after realignment are interpreted as bona fide sources from the sample (red circles in Fig. 1g and h). This procedure was applied to all magnetic field maps in our study.

Once a signal was identified at the identical location in both repeat magnetic field maps, analyzing magnetic parameters of the underlying source can provide further evidence as to whether the source was within the sample or on its surface. We computed best-fitting dipole models for each source using a least-squares inversion algorithm, which yielded all six parameters for a point magnetization source: the three-dimensional location of the source and its three-dimensional magnetization vector (see Experimental section).^16,34^ By subtracting 1 µm from the computed map-to-source distance to account for the thickness of the magnetic field sensitive layer in the diamond, we obtain the depth of the source from the diamond surface. Given the minimal topography of our embedded and polished samples, which enables a distance between sample and diamond of less than 1 µm, we considered sources greater than 1 µm from the diamond to lie within the sample. In addition, samples were exposed to a strong magnetic field in the plane of the thin section towards the 12 o'clock direction (0° declination, 0° inclination) prior to measurements (Fig. 1i). Only sources whose magnetizations aligned toward this direction of the applied magnetic field can be considered bona fide.

**Fig. 2:**
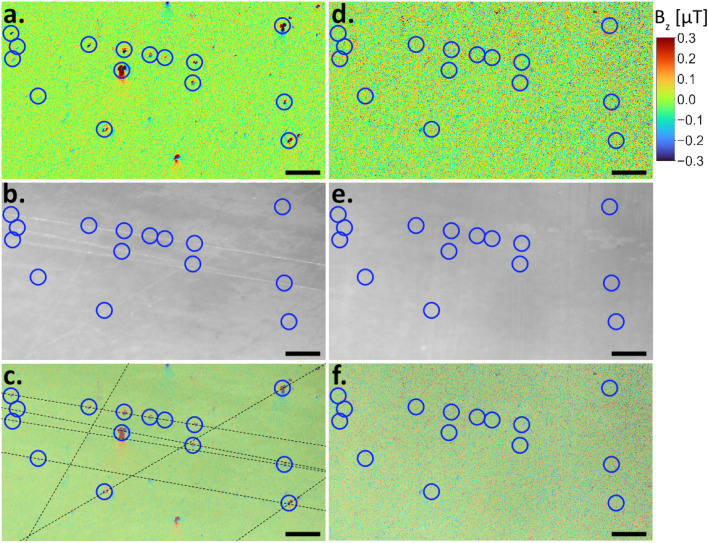
Contamination detected within microscopic scratches (a–c) and their removal after polishing (d–f). (a) Magnetic field map before polishing with multiple dipolar signals (some marked with blue circles). (b) Corresponding reflected light image with well-visible scratches. (c) Magnetic field map superimposed on reflected light image showing the correlation of signals within scratches (highlighted by black lines). (d) Magnetic field map showing the removal of dipolar signals after polishing. Blue circles mark the location of signals found before polishing; dipolar signals are no longer observable. (e) Scratches were also removed by polishing. (f) Superimposed map and reflected light image. Scale bars: 100 µm.

A final way to identify if the source of a signal was contamination was the potential co-occurrence with artifacts on the sample surface visible in the reflected light images. We initially polished the samples with coarse grit sandpaper to remove surface layers. Polishing also facilitated optimal coupling with the QDM diamond sensor, yet microscopic scratches often remained on the sample surface. These micro-scratches regularly trapped contaminating particles (Fig. 2). We therefore extensively polished the samples before collecting a QDM map pair using non-magnetic, 1 µm alumina grit until no scratches were visible in both light microscopy and the reflected light image of the QDM (see Experimental section) (Fig. 2).

### Human brain tissue

Human brain samples (Table S1) were embedded in methyl-methacrylate (MMA) at LMU Munich (Munich, Germany) following established protocols.^5,35^ No sample preparation step, including formalin-fixation, should have impacted the stability of iron-oxide nanoparticles in the tissue.^9^ To avoid contaminating magnetic particles,^13^ all chemicals were filtered using 50 nm pore sizes, except for MMA, where pore sizes of 200 nm were used due to its higher viscosity. All glassware and plastic containers were acid cleaned and washed with filtered distilled water. We cut thin sections from embedded human brain samples (approximately 8 cm^3^) and removed potential contaminants from the saw that was used for cutting by polishing off the upper 200 µm of each thin section (see Experimental section).

We collected a total of 13 map pairs from eight different human brain samples: seven from the mesencephalon and one from the medulla oblongata (Table S1). All samples were magnetized with a 1.5 T pulse field in the plane of the thin section prior to image collection. Most FOVs were selected based on the presence of neuromelanin-containing cells (Fig. 1a–c) to facilitate the alignment of duplicate maps. Neuromelanin is a pigment produced by specific neurons in the brain stem, most likely to shield cells from redox active metals, toxins, and catecholamines.^36^ It is mainly present in the substantia nigra pars compacta (SNpc), but neuromelanin-containing cells can be found throughout the brain stem.^36,37^ We used these cells for sample alignment since they were clearly visible in the QDM reflected light images.

**Fig. 3:**
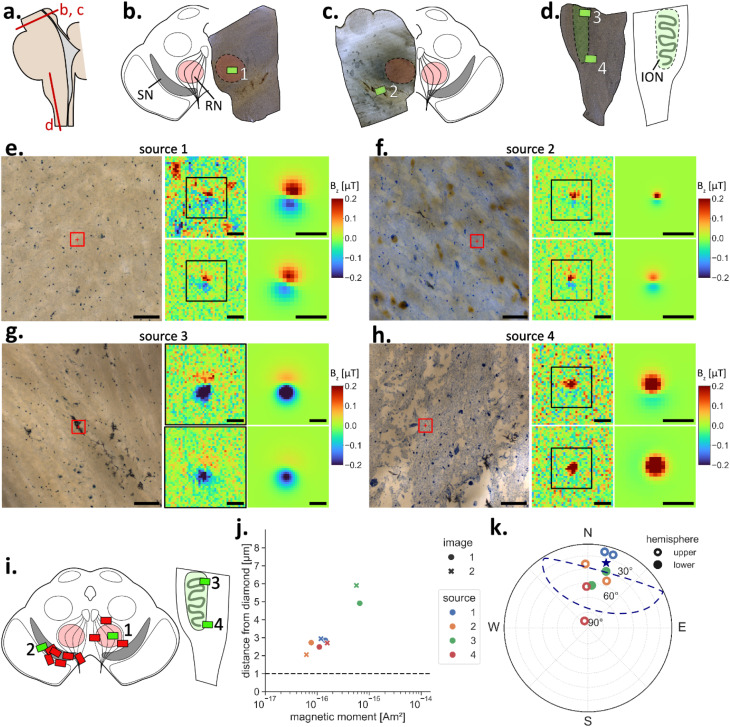
Magnetic dipoles detected in human brain tissue with the QDM. (a) Drawing of a human brain stem illustrating the origin of the samples (red lines) in the mesencephalon (b and c) and the medulla oblongata (d). (b–d) Photographic images of the samples with corresponding drawings showing the locations of the QDM FOVs (green rectangles) on the sample's surfaces, in which sources were identified. The FOVs were collected over (b) the red nucleus (RN, red), (c) substantia nigra (SN, gray), and (d) the inferior olivary nucleus (ION, green). (e–h) For each identified source, photomicrographs marking the location of the source (red squares), both signals in the repeated magnetic field maps (image 1 and 2), as well as the corresponding dipole model fits are shown. (i) Drawings illustrating the positions of all FOVs collected on human brain tissue samples. FOVs hosting identified sources indicated by green rectangles (*n* = 4); red rectangles indicate FOVs where no sources were identified (*n* = 9). (j) Distance from diamond *versus* magnetic moment from dipole model fits for each source and image. All four sources were within the tissue since they lie >1 µm away from the diamond (horizontal line). (k) Magnetization directions of the dipoles. The mean direction (blue star) and 95% confidence ellipse (blue line) indicate that the direction of the initial magnetizing field (declination = 0°, inclination = 0°) coincided with the magnetization direction of the sources (open symbols upper hemisphere, solid lower hemisphere). Scale bars: 100 µm in photomicrographs; 10 µm in magnetic field maps.

We identified dipole signals from four sources in three human brain samples that met the criteria of both being in tissue and being aligned towards the northern hemisphere (Fig. 3). The four sources were found in the red nucleus (RN), the SNpc, and the inferior olivary nucleus (ION) (Fig. 3). All four particles were detected in small local nerve fibers but could not be assigned to specific neurons. This argues against the hypothesis of magnetite particles serving a physiological function (*e.g.*, magnetic field sensing^1^), since such particles should be expected to reside in neuron perikaryons. However, with only four identified sources, this conclusion is tentative. One source was found adjacent to a light scattering, opaque structure in brightfield microscopy (Fig. 3g), which was most likely a bubble that formed during polymerization. Polymerization should not introduce magnetic contaminants to a sample, since the compounds in the resin cannot combine to form magnetic materials. However, this source should be regarded with some caution due to its proximity to the bubble.

### Rat brain tissue

In addition to human brain tissue, we collected 14 QDM map pairs from four rat cerebral cortex samples (Table S2). Samples came from four animals held in a vivarium adjacent to a heavily trafficked tunnel.^38^ Three of the animals were exposed to unfiltered traffic-related air pollution, while one animal was provided with filtered air (#4999). Cortex samples (2 mm thick coronal tissue blocks) were embedded in Epon resin at Harvard Medical School (Boston, USA). Epoxy blocks containing embedded rat brain samples were cut in half, then fixed onto the QDM sample holder after polishing. Due to the lack of neuromelanin-containing cells, all rat brain samples were stained with filtered toluidine-blue prior to image collection. Surface areas of rat brain tissue (0.7–2.8 mm^2^) were less than the FOV of the QDM (3.2 mm^2^), so blank epoxy surrounding the tissue was also imaged. Subsequent sample preparation, QDM image collection and signal identification used the same techniques as for the human brain tissue. We imaged up to three FOVs per rat brain sample. Two samples were repeatably imaged and polished, removing several micrometers of tissue in between collecting map pairs, to investigate potential changes with depth (up to five polishings/sample; Table S2).

**Fig. 4:**
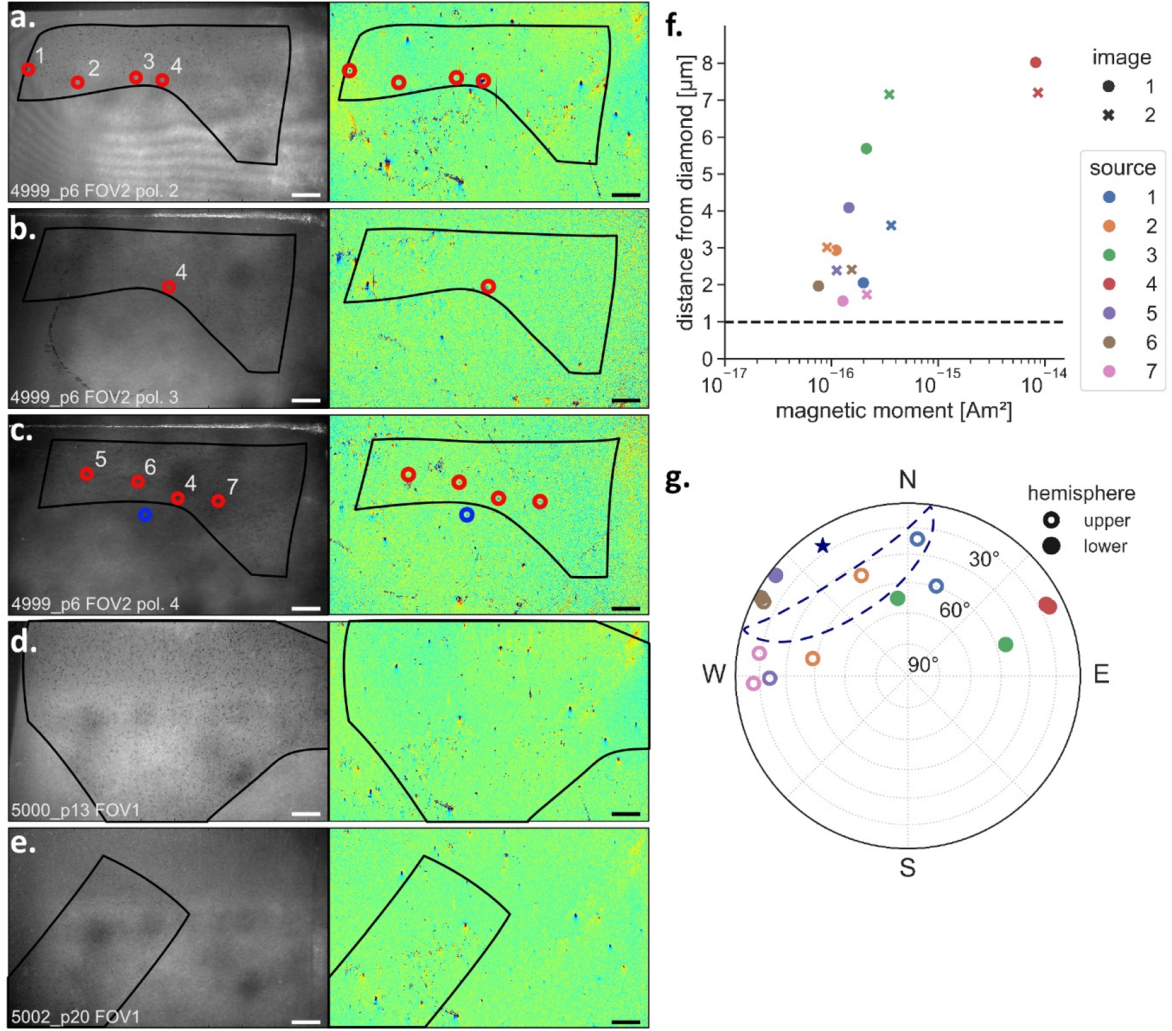
Sources detected in magnetic field maps of rat brain samples meeting the selection criteria. (a–e) Reflected light images and magnetic field maps from five FOVs of rat brain tissue that resulted in valid QDM images. The first (image 1) of the two, repeat QDM images are shown. Nine sources (red circles) were found in sample 4999_p6 (a–c), while none were found in two other samples (d and e). One source was situated in blank epoxy and not in rat tissue (blue circle). Source #4 was detected in all three FOVs of sample 4999_p6 and therefore treated as one source, decreasing the total number of detected sources to seven. (f) Distance from diamond plotted against the magnetic moment for each source and image. The horizontal dashed line indicates the cut-off distance >1 µm from the diamond. (g) Magnetization directions of detected sources. The mean direction (blue star) and confidence ellipse (blue line) contain the direction of the initial magnetizing field (declination = 0°, inclination = 0°) within 95% confidence limits (open symbols upper hemisphere, solid lower hemisphere). Scale bars: 200 µm.

Of the 14 collected map pairs, four showed high numbers of sources in blank epoxy (Fig. S1). We interpreted these signals as surficial contamination and discarded the maps from further analysis. Analyzing the directions of magnetization of the remaining identified sources revealed that dipoles in the map pairs from sample 4998_p5 clustered at two antipodal directions with declinations of ∼120° and ∼300° (Fig. S2). We tentatively interpret this as a consequence of undesired sample motion during data acquisition, which resulted in a smearing effect of the magnetic dipole sources. In any case, we did not observe this issue in any other map, so we excluded maps from this sample from further analysis. Applying our selection criteria on the sources identified in the remaining five map pairs (Fig. S2) led to nine sources detected in three magnetic field map pairs within tissue (Fig. 4). The nine sources met the criteria of lying >1 µm from the diamond (Fig. 4f) and had northerly directions consistent with the bias magnetic field (Fig. 4g). These are therefore considered as bona fide sources in rat brain tissue. The nine sources were found in three repeated FOVs of one sample (4999_p6), while two other samples (5000_p13 and 5002_p20) contained no detectable sources (Fig. 4).

One additional source was situated in blank epoxy that surrounded the tissue (blue circle, Fig. 4c). All other bona fide sources in the rat brain samples were within tissue. According to binomial tests, in which we used the ratio of tissue area to FOV area as the hypothesized probability to detect a source in tissue (*i.e.*, we tested whether sources were placed randomly over the FOV or not), there was a statistically significant preference of sources to be in tissue and not in epoxy. This was the case in the two map pairs that showed several sources, while a similar test for the third map of 4999_p6 was not significant as it had only a single source (Table S3). This is additional evidence that the sources detected in tissue were genuine and not contaminants. The source outside of tissue was not considered in further analysis.

One source was found at the same location and with a similar magnetization direction in each of the three repeated FOVs of sample 4999_p6 (source #4 in Fig. 4) and was therefore the same source imaged three times. The magnetic dipole moment of this source was similar in the first two maps (8.2 × 10^−15^ Am^2^ and 1.3 × 10^−14^ Am^2^), which was the strongest magnetic moment of all sources, but then decreased in the third map (5.3 × 10^−16^ Am^2^). The signal was likely generated by several closely packed magnetite particles, since such a strong signal could not have been generated by an individual single-domain magnetite particle. The lower magnetic moment in the third map likely resulted from the removal of particles by polishing. Seven sources were therefore identified in the control sample from an animal that was breathing filtered air. We identified no sources in samples from rats held in a polluted atmosphere (5000_p13 and 5002_p20). This is the opposite of what one would expect if pollution was the source of magnetite nanoparticles in brain tissue,^2^ noting that this observation is based on only five QDM map pairs from three animals.

### Magnetic sources in brain tissue

Magnetic moments retrieved from dipole models of the detected sources ranged in human brain tissue from 6.2 × 10^−17^ to 6.6 × 10^−16^ Am^2^ (Fig. 3j) and in rat brain tissue from 7.5 × 10^−17^ to 1.3 × 10^−14^ Am^2^ (Fig. 4f). Recalling that the QDM samples from human brain tissue were obtained from large (8 cm^3^) pieces, and that the magnetization of the pieces were measured prior to the QDM study,^5^ we calculated the magnetic moment expected to be present in human tissue corresponding to the volume of the QDM image, assuming a homogeneous distribution of magnetic particles and a detection depth of 6 µm, as all sources in human brain tissue were within 6 µm. The total moments measured by the QDM were one to two orders of magnitude less than expected (Fig. S3). Moreover, sources were only detected in four of 13 FOVs (Fig. 3i). For rat brain tissue, the magnetizations of the original brain samples were not measured, which prevents a similar calculation. With respect to the imaged area, we detected significantly more sources in rat brain tissue compared to human brain tissue (4 sources in ∼41 mm^2^ of human tissue *versus* 7 sources in ∼7 mm^2^ of rat tissue). Whether this was due to a physiological difference between tissues remains unclear, since the samples originated from different laboratories with different sample preparation techniques. However, even for rat brain samples, we detected only seven sources in five FOVs (Fig. 4a–e). Three possibilities can explain the lack of sources in the magnetic field maps of brain tissue: (i) QDM sample preparation, such as embedding in MMA, may have chemically altered the magnetite. (ii) Magnetite in brain tissue might be clustered, *e.g.*, not homogeneously distributed, and the QDM images missed the clusters. (iii) The majority of magnetite particles had moments below the detection threshold of the QDM.

To help distinguish these three possibilities, we prepared a sample of magnetotactic bacteria (MTB), which grow single domain sized magnetite crystals in chains, using the same chemicals and protocol as used for the tissue samples (see Experimental section). The MTB sample yielded strong magnetic signals (Fig. S4), which excludes the possibility that the embedding process chemically altered the magnetite. Bulk magnetic measurements of brain tissue show evidence for magnetic interactions,^2,13,14,39,40^ which support clustering of magnetite particles. Such clusters have been observed in TEM images of brain sections *in situ*.^2^ On the other hand, TEM imaging also documented isolated, single crystals.^2,8^ Source #4 in Fig. 4 from rat brain tissue was most likely generated by a cluster of magnetite particles. However, our QDM data found only one such cluster in 18 FOVs covering a total area of ∼48 mm^2^ of tissue. We therefore consider the presence of clustering to partly but not fully explain the small number of sources.

The detection threshold of the QDM in high-sensitivity mode applied to speleothems (calcium carbonate-rich cave precipitates) was *ca.* 1 × 10^−16^ Am^2^.^32^ Those maps were acquired using ∼35-minute integrations, whereas we integrated for up to 12 hours to collect a single magnetic field map. In principle, this longer integration should reduce the noise floor by a factor of 4–5. To quantify the noise level of the QDM under the conditions of the present study (high-sensitivity mode, ∼12-hour acquisition), we selected the smallest resolvable dipolar signals in the magnetic field maps, regardless of their origin (*e.g.*, including potential contamination). Based on the magnetic moment of 147 of the smallest signals across eleven maps, most sources were on the order of 4 × 10^−17^ Am^2^ (Fig. S5). Although we were able to fit some weaker sources (minimum 8 × 10^−18^ Am^2^), we adopt 3 × 10^−17^ Am^2^ as a conservative threshold for our measurement scheme, consistent with previous estimates of the theoretical QDM resolution (∼1 × 10^−17^ Am^2^).^16^ Assuming individual, spherical, non-interacting magnetite with a saturation magnetization of 480 kA m^−1^,^41^ a magnetic moment of 3 × 10^−17^ Am^2^ corresponds to a particle diameter of approximately 50 nm – close to the superparamagnetic threshold of ∼30 nm at room temperature.^42^ This moment sensitivity exceeds that of other magnetic microscopy techniques, such as SQUID microscopy (∼1 × 10^−15^ Am^2^), significantly.^16,34^ While others recently achieved a magnetic moment sensitivity <1 × 10^−18^ Am^2^ using an NV-center approach, their FOV (4.4 µm) was limited, restricting applicability for nanoparticle detection in extended samples.^43^

**Fig. 5:**
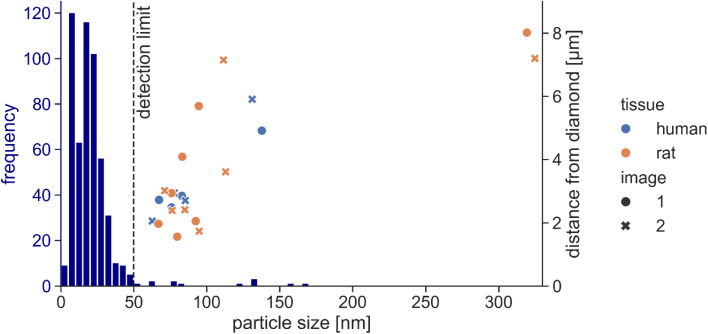
Distance from the diamond sensor as a function of particle size for sources detected in both human and rat brain tissue. Results from both images (1 and 2) are shown for each source. The size distribution from a TEM imaging study^2^ (blue colored histogram) illustrates the expected particle sizes in brain tissue. Most magnetite particles are below the QDM detection limit of 50 nm as defined in our study. Most particles detected with the QDM match the rare occurrence of larger particles in TEM images. One strong signal in rat brain tissue (particle size above 300 nm) was most likely from a cluster of smaller particles.

Magnetic moments of most individual sources detected in brain tissue corresponded to particle sizes of 63–138 nm (Fig. 5). One strong signal in rat brain tissue with a theoretical particle size above 300 nm originated most likely from a cluster of smaller particles. Based on the noise characterization, we can conclude that the magnetic moment of bulk brain samples likely arises mainly from magnetite particles that are smaller than 50 nm and only a fraction of the total magnetic moment of brain tissue originates from larger particles. So far, only TEM imaging studies have quantified magnetite particle sizes in brain tissue. One study found an average diameter of 33.4 ± 15.2 nm, with some particles as large as 90–200 nm,^1^ while particles in another study ranged from 5 to 30 nm,^15^ and a third based on 533 extracted and imaged magnetite particles found median sizes of 18 and 14 nm along the long and short axes, while some particles reached up to 150 nm (Fig. 5).^2^ The signals we identified with the QDM coincide with the larger magnetite particles observed in TEM micrographs (Fig. 5). Our results and the lack of magnetic moment in the QDM maps therefore seem to reflect a low concentration of larger magnetite particles in brain tissue while magnetite particles in most of the size range remain below the detection threshold.

Magnetic particles and airborne dust pollutants are ubiquitously present in the environment,^4,44^ underlying the importance to maintain a sample preparation environment free from magnetic contamination and to quantify any contaminant sources that exist. The QDM offers the unique ability to distinguish between surface contaminants and bona fide sources through multiple measures. First, two repeat images should be collected for each FOV, cleaning the sample and diamond sensor surfaces prior to image collection. Second, assuming the samples were in close contact with the diamond (<1 µm), sources lying >1 µm from the diamond likely reside within tissue. Third, the magnetization directions should be biased towards the direction of the magnetic field applied to the sample prior to QDM imaging. Sources that fulfill these criteria likely originate from tissue. The ability to differentiate bona fide sources from contamination is a unique feature of the QDM, allowing a non-destructive detection of magnetic particles in tissue that helps identify their precise anatomical relationship with surrounding cells.

## Conclusions

We demonstrated the capability to detect individual single-domain magnetite particles down to 50 nm diameter in a wide-field QDM with a millimeter-range FOV. We achieved this by operating the QDM in a high-sensitivity mode coupled with long acquisition times. In addition, by collecting repeat, slightly shifted magnetic field maps of the same location, we obtained the ability to distinguish magnetic signals that originate from bona fide sources within a sample and those that stem from artifacts from the diamond sensor, noise, or surface contamination. Dipole models fit to the detected signals provide quantitative moments and directions thereby offering a unique capability to detect and image magnetic signals from magnetite nanoparticles within tissue samples. While the presence of magnetite particles in tissue is well-known, pin-pointing their precise location remains challenging due to the restricted FOVs inherent to electron microscopy. The duplicate, high-sensitivity magnetic microscopy images from the QDM allowed us to identify magnetite nanoparticles in brain tissue. To our knowledge, this study is the first to present magnetic dipole imaging of individual, single-domain magnetite particles in brain tissue. The sources identified in human and rat brain samples span a size range of 60–135 nm, consistent with the larger end of the particle size distribution observed by TEM analyses of brain samples. Our results show that, while the majority of magnetite particles in brain tissue are smaller than 50 nm, larger particles in low concentrations can be identified with quantum sensing techniques. Further studying the exact location and magnetic properties of magnetite particles within brain tissue using the QDM will help understanding their physiological implication for the brain.

## Experimental section

### Samples

#### Human brain samples

The collection of human brain tissue samples has been previously described.^5^ In short, human brain stems were obtained during autopsy at the University of Rostock (Germany) in accordance with relevant guidelines and regulations in Germany and approval from the Local Ethical Committee at the Medical University of Rostock (ethical approval number: A 2021-0282). Forensic autopsies were performed as requested by the public prosecutor and approved by a judicial decision. For this type of autopsy, formal consent is not required. Sample extraction was conducted using acid-cleaned ceramic tools to avoid the risk of contamination. Immediately after extraction, the brain stems were stored in sterile plastic bags at −20 °C. We fixed four brain stems with 10% formalin for a period of five days. Following fixation, we removed the dura and arachnoid mater as well as large blood vessels, and then cut the brain stems into six samples (medulla oblongata, pons, and mesencephalon of each hemisphere). Table S1 provides details about the samples investigated in the present study.

#### Magnetotactic bacteria

We extracted MTB from a freshwater pond in Bavaria, Germany. A pure MTB sample was created using a MTB enrichment method,^45^ in which all other organisms or particles were filtered out. We confirmed the presence of MTB by light microscopy imaging and identified different MTB morphotypes including cocci, spirilla, and rod-shaped MTB.

#### Rat brain samples

Four cerebral cortex samples were obtained from four animals (Table S2). All animal procedures have been previously described^38^ and were performed in compliance with protocols approved by the University of California Davis (UC Davis) Institutional Animal Care and Use Committee (IACUC), with the goal of minimizing pain and suffering. Three samples were from animals housed from 1 month in age in a vivarium adjacent to a heavily trafficked tunnel in northern California and thus exposed to gases and particulates derived from traffic-related air pollution. Samples #5000 and #5002 were from female, 15 months, transgenic rats; #4998 from a female, 3 months, transgenic rat. Sample #4999 was from a female, 15 months transgenic rat, housed in an adjacent vivarium but exposed only to filtered air. For more details, see original publication.^38^

### Magnetometry

We measured magnetic moments of bulk human brain samples with a 2G Enterprises, Inc. (Mountain View, CA, USA), Model 755-4K, superconducting rock magnetometer following existing protocols.^3^ The data were previously reported.^5^

### Sample preparation for QDM

#### Human brain samples

We embedded both mesencephalon samples and one medulla oblongata sample from each of four human brain stems in MMA (Table S1). Embedding was conducted in accordance with existing protocols^5,35^ by dehydrating the samples with successive ethanol baths of increasing concentration, degreasing with xylene, and incubating in methanol, with a minimum of three days of incubation per step. The samples were then transferred into liquid MMA mixed with dibutyl phthalate and benzoyl peroxide, which became polymerized in a controlled environment within 14 days. Chemicals were filtered with PTFE filters (50 nm mesh size; viscous chemicals were filtered with a 0.2 µm mesh size) to minimize potential magnetic contamination. Glassware and containers were washed with 10% HCl and filtered distilled water. We cut slices of approximately 600 µm thickness from the polymerized MMA blocks using a circular saw microtome (SP 1600, Leica, Wetzlar, Germany). The slices were ground and polished with a 400 CS micro-grinder (EXAKT Advanced Technologies, Norderstedt, Germany), which decreased the slice thicknesses to approximately 400 µm. Surfaces were thoroughly cleaned with isopropanol after polishing. Preceding QDM measurements, we polished the samples' surfaces for a minimum of one minute using alumina grit (MicroPolish Alumina 1 µm, Buehler, Lake Bluff, IL, USA), cleaned the samples in an ultrasonic cleaner with Milli-Q water for one minute (Ultrasonic Cleaner, VWR International, Radnor, PA, USA), and wiped the surfaces with isopropanol. This was repeated if any scratches were found on the samples' surface in light microscopy images. We subjected the samples to a 1.5 T pulse field in the in-plane direction of the thin sections that corresponded to 0° declination and 0° inclination in QDM coordinates.

#### Magnetotactic bacteria

The MTB sample was prepared using the same protocol as for tissue samples, except for the xylene step. Since the enriched MTB formed a small pellet of *ca.* 2 mm diameter, the cells were incubated in each chemical for 10 minutes instead of three days. For final polymerization, 30 µl of MTB in liquid MMA was placed in a hole (3 mm diameter, 2.5 mm depth) that was drilled into a pre-polymerized block of blank MMA with a titanium nitrite coated drill bit. Following polymerization, thin sections were cut from this MMA block and polished in the same manner as the human tissue samples.

#### Rat brain samples

Tissue samples were cut into 2 mm thick coronal tissue blocks and post-fixed in buffered 4% PFA for 24 hours. Once fixed, the blocks were cryoprotected in buffered 30% sucrose for several days and then frozen in OCT compound for cryosectioning. We washed the OCT embedded rat brain samples with filtered distilled water, dehydrated them in filtered acetone, and infiltrated them with a mixture of filtered acetone and Epon (TAAB Laboratories Equipment Ltd, https://taab.co.uk) overnight. The samples were subsequently transferred into 100% Epon and left to polymerize at 60 °C for 48 hours. All preparation steps for rat brain samples were done in HCl-washed glass scintillation vials. We cut the embedded rat brain samples in half using an HCl-washed ceramic knife and polished the surfaces using alumina grit (MicroPolish Alumina 1 µm). The samples were exposed to a 1.5 T pulse field in the in-plane direction of the thin sections (0° declination and 0° inclination). We subsequently stained the rat brain samples with filtered toluidine-blue prior to image collection to facilitate sample alignment on the QDM.

### Quantum diamond microscope

The QDM, developed and built at Harvard University, is housed in a magnetically shielded room within a clean room environment that minimizes the risk of surface contamination on the samples and the diamond sensor. Each time before loading a sample on the QDM, the diamond sensor was removed and carefully cleaned with acetone and isopropanol. The samples were cleaned solely with isopropanol, since acetone can damage MMA/resin. For sample alignment, the QDM recorded a reflected light image with a bright-field LED that uses the same optics as for magnetic field imaging. Therefore, magnetic field maps and reflected light images of one QDM image had the exact same FOV. The choice of location for each QDM image was based on recognizable features in the reflected light image, such as neuromelanin-containing cells.

We operated the QDM in a high-sensitivity mode. Microwave mixing was used to resonate with both ^15^N hyperfine states of NV-centers. These states lead to fluorescence peaks that are separated by the hyperfine parameters of ∼3 MHz.^31^ Each measurement comprised between six and 22 iterations, each with an integration time of ∼35 minutes. The iterations were stacked and averaged to reach high signal-to-noise ratios. The direct-current bias field was kept at 0.9 mT and was reversed repeatedly during the measurements to achieve a near-zero net bias field cancelling induced magnetizations. With this setup, the resulting map represents remanent magnetization in a 300 nT bias field. The MTB sample was imaged with the QDM in normal operation mode with a single iteration. We operated the QDM in projective magnetic microscopy (PMM) mode, which results in raw magnetic field maps that reveal the magnetic field strength in the 〈111〉 crystallographic direction. The magnetic field component perpendicular to the surface (*B*_*z*_) was computed from the raw magnetic field maps using spectral methods.^34^ To correct for global fluorescence, we used a fraction of 0.25 for the non-local out of the global fluorescence.^16^ The FOV of the QDM was 1920 × 1200 pixels, which translates to 2.25 × 1.40 mm^2^ given a pixel size of 1.17 µm. Pixel binning was not performed to achieve the highest possible spatial resolution. Data acquisition and analysis was done with QDMlab.^46^

### Dipole modelling

Dipole model fits were computed based on a least-squares inversion algorithm.^16,34^ From the dipole model fits for each detected signal, we retrieved magnetic moments, distances between source and sensor, and orientations of the source (declination and inclination) using QDMlab.^46^ Since the magnetic field sensing NV center layer in the diamond chip had a finite thickness, we subtracted 1 µm from the distance between source and sensor. Particle sizes were computed assuming spherical, non-interacting magnetite particles with a saturation magnetization of 480 kA m^−1^.^41^

### Light microscopy

We stained human brain samples with toluidine blue for light microscopy and acquired images with a light microscope (Axio imager.M2, ZEISS, Oberkochen, Germany), equipped with a 40× objective (Plan-Apochromat 40×, ZEISS, Germany) and operated with Stereo Investigator software (MBF Bioscience, Williston, VT, USA). Microscopy images were analyzed using an image browser (Biolucida Viewer, MBF Bioscience) and a raster graphics editor (Affinity Photo 2, Serif Europe Ltd, Nottingham, UK). Only contrast and brightness were adjusted, without altering the appearance of the original materials.

### Statistics

Directional mean and corresponding confidence ellipses were calculated with Fisher statistics using PaleoMac software.^47^ One-tailed, right-sided binomial tests with the ratio of tissue area over FOV area as hypothesized probability of success were used to test for a preferential location of sources in QDM maps of rat brain samples.

## Author contributions

Leon Kaub: conceptualization, methodology, validation, formal analysis, investigation, data curation, writing – original draft, visualization; Stuart A. Gilder: conceptualization, methodology, validation, resources, writing – review & editing, supervision, project administration, funding acquisition; Roger R. Fu: conceptualization, methodology, software, validation, formal analysis, investigation, resources, data curation, writing – review & editing, supervision; Barbara A. Maher: methodology, resources, writing – review & editing; Gabriel Maxemin: investigation; Aaron T. Kuan: investigation, formal analysis, writing – review & editing; Andreas Büttner: resources; Stefan Milz: methodology, investigation, resources, writing – review & editing; Christoph Schmitz: conceptualization, validation, resources, writing – review & editing, funding acquisition.

## Conflicts of interest

There are no conflicts to declare.

## Supplementary Material

RA-016-D5RA08546B-s001

## Data Availability

Data types for this work consist of raw QDM magnetic field maps, maps of the vertical component of the magnetic field, and reflected light images. The data that support the findings of this study are openly available in Zenodo at https://doi.org/10.5281/zenodo.14958851, reference number 14958851.^48^ Supplementary information (SI): tables and figures that are already described and referenced in the main text. See DOI: https://doi.org/10.1039/d5ra08546b.
