# Supplementary material for: Magnetic dipole imaging of magnetite nanoparticles in brain tissue
Source: RSC Adv. 2026 Jan 5;16(2):983–94. doi: 10.1039/d5ra08546b (PMC12766265; doi:10.1039/d5ra08546b)
Supplement: RA-016-D5RA08546B-s001 [file RA-016-D5RA08546B-s001.pdf]

Supplementary Information (SI) for RSC Advances.
This journal is © The Royal Society of Chemistry 2025

Supplemental Material

Magnetic Dipole Imaging of Magnetite Nanoparticles in Brain Tissue

Leon Kaub[a,b], Stuart A. Gilder[a], Roger R. Fu[c], Barbara A. Maher[d], Gabriel Maxemin[c], Aaron T. Kuan[e], Andreas Büttner[f], Stefan Milz[b], and Christoph Schmitz[b]*

[a] Dept. of Earth and Environmental Sciences, LMU Munich, Theresienstr. 41, 80333 Munich, Germany

[b] Dept. of Anatomy II, Faculty of Medicine, LMU Munich, Pettenkoferstr. 11, 80336 Munich, Germany

[c] Dept. of Earth and Planetary Sciences, Harvard University, 20 Oxford St, Cambridge, MA 02138, USA

[d] Centre for Environmental Magnetism & Palaeomagnetism, Lancaster University, Lancaster, LA1 4YQ, UK

[e] Dept. of Neuroscience, Yale School of Medicine, 333 Cedar St, New Haven, CT 06510, USA

[f] Institute of Forensic Medicine, Rostock University Medical Center, St.-Georg-Str. 108, 18055 Rostock, Germany

* Corresponding author: leon.kaub@lmu.de

***Table S1**. Details on human brain samples imaged with the QDM.*

case ID	age [yrs]	sex	T_{pm} [hours]	cause of death	section	hemi-sphere	sample	FOV
141/22	66	female	69	LC	mesencephalon	right	3974_3	2
141/22	66	female	69	LC	mesencephalon	right	3974_3	3
141/22	66	female	69	LC	mesencephalon	right	3974_5	1
141/22	66	female	69	LC	mesencephalon	right	3974_5	2
141/22	66	female	69	LC	mesencephalon	left	3975_2	1
141/22	66	female	69	LC	mesencephalon	left	3975_3	1
141/22	66	female	69	LC	mesencephalon	left	3975_3	2
141/22	66	female	69	LC	medulla oblongata	left	3976_1	1
141/22	66	female	69	LC	medulla oblongata	left	3976_1	2
144/22	61	male	45	MI	mesencephalon	right	3977_2	1
144/22	61	male	45	MI	mesencephalon	right	3977_2	2
157/22	74	female	9	MI	mesencephalon	left	3982_3	3
159/22	56	male	41	HS	mesencephalon	right	3985_2	1

Causes of death: LC, liver cirrhosis; MI, myocardial infarct; HS, hemorrhagic shock. T_{pm}, post-mortem interval; FOV, field-of-view.

Table S2. *Details on rat brain samples imaged with the QDM. Rats 4998, 5000, and 5002 were held in polluted atmospheres, while rat 4999 was exposed to filtered air.*

rat ID	age [months]	sex	polluted air [months]	region	sample	FOV	Polish	tissue area [mm^2]
4998	3	female	2	cortex	4998_p5	1	1	0.77
4998	3	female	2	cortex	4998_p5	1	2	0.75
4998	3	female	2	cortex	4998_p5	1	3	0.78
4998	3	female	2	cortex	4998_p5	1	4	0.82
4998	3	female	2	cortex	4998_p5	1	5	0.72
4998	3	female	2	cortex	4998_p5	2	1	2.77
4998	3	female	2	cortex	4998_p5	2	2	2.78
4998	3	female	2	cortex	4998_p5	3	1	1.28
4999	15	female	0	cortex	4999_p6	2	1	1.26
4999	15	female	0	cortex	4999_p6	2	2	1.12
4999	15	female	0	cortex	4999_p6	2	3	1.15
4999	15	female	0	cortex	4999_p6	2	4	1.04
5000	15	female	14	cortex	5000_p13	1	1	2.55
5002	15	female	14	cortex	5002_p20	1	1	0.95

FOV, field-of-view.

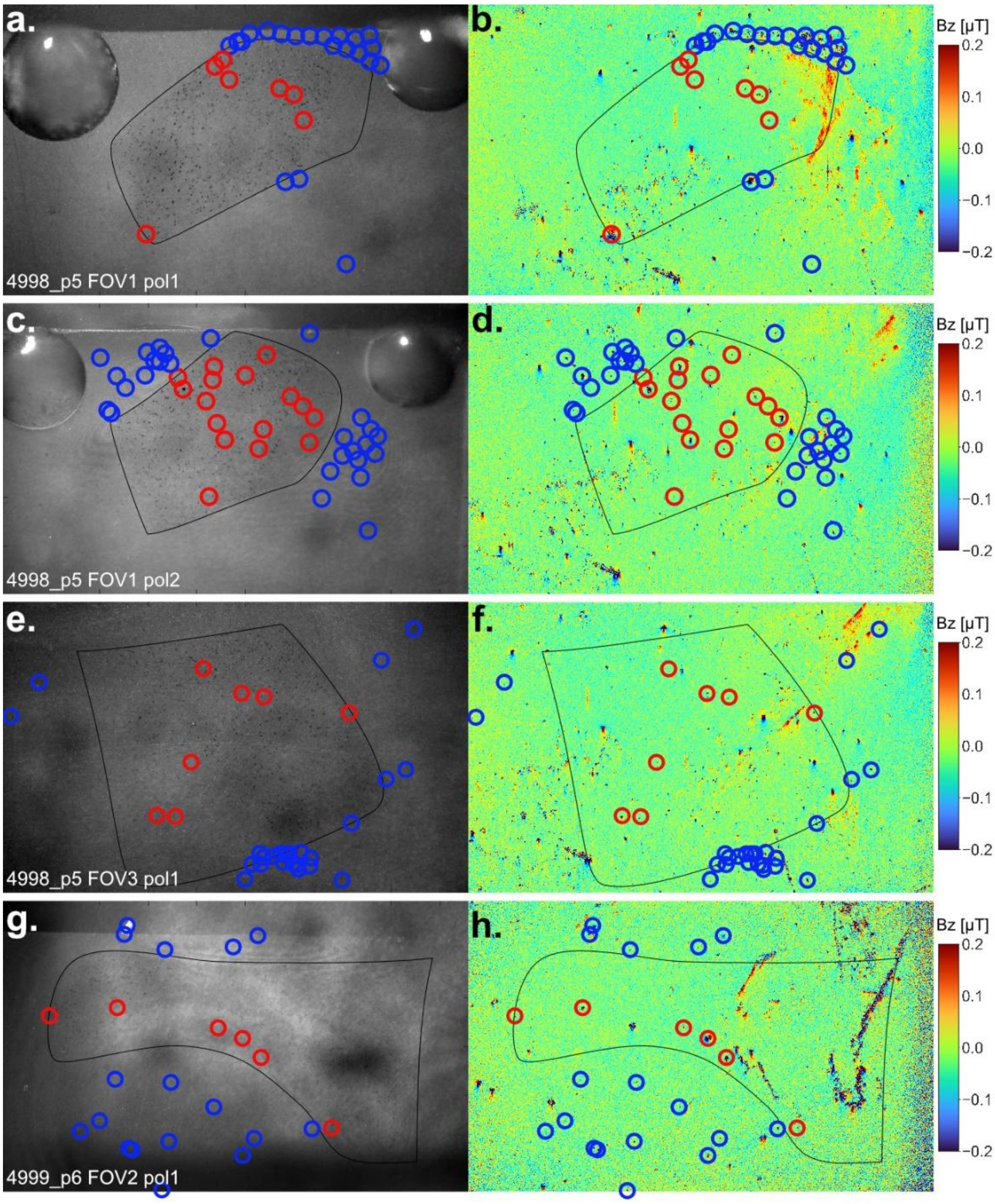

Fig. S1. *Surface contamination observed in four different rat brain FOVs (only one image shown for each map pair). Reflected light images (**a**, **c**, **e**, **g**) and magnetic field maps (**b**, **d**, **f**, **h**) from the QDM map pairs show high numbers of sources in blank epoxy (blue circles). In these samples, relatively high numbers of sources were also identified in tissue (red circles) compared to other map pairs.*

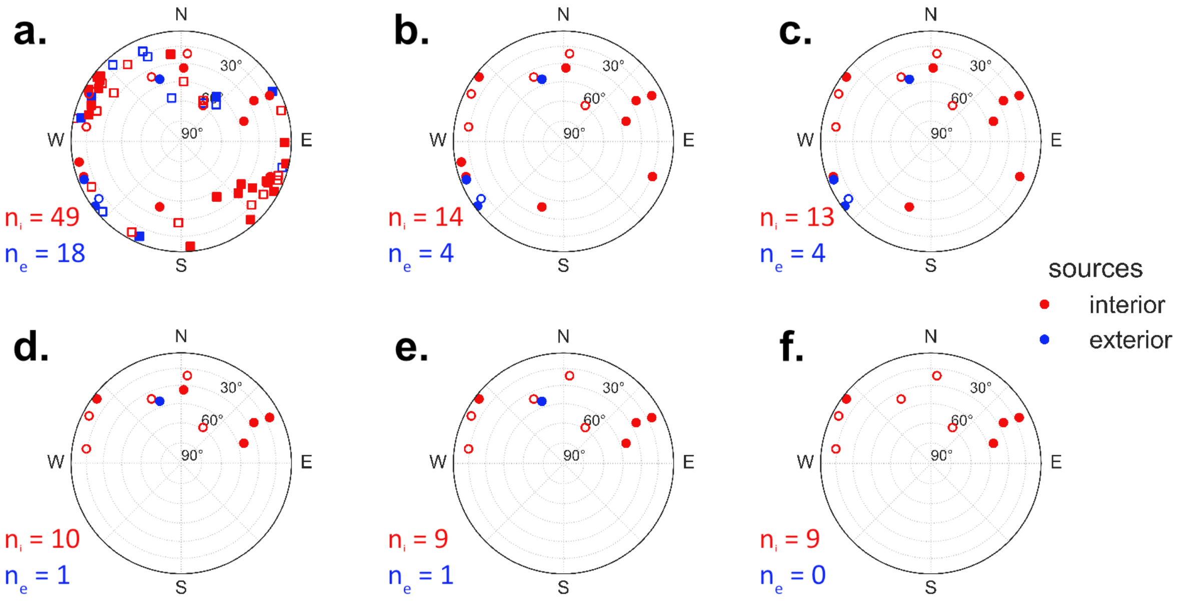

Fig. S2: *Direction of magnetization of sources identified in rat brain tissue.* ***a****) All sources that were identified in repeat magnetic field maps, divided into interior (red, tissue) and exterior (blue, blank epoxy) sources. The effect of removing sources based on defined selection criteria is reflected in the decreasing number of sources (n_i, number of interior sources; n_e, number of exterior sources). Sources from sample 4998_p5 (squares) clustered in two antipodal directions at ~120° and ~300°.* ***b****) All sources after removing those from 4998_p5.* ***c*** *and* ***d****) Directions after applying the criteria that all sources need to be > 1 µm from the diamond (c) and aligned towards the northern hemisphere (d).* ***e****) One source had a different direction of magnetization in each of the two repeated magnetic field maps and was omitted.* ***f****) The final, interior sources met all selection criteria and were therefore considered bona-fide sources. Note that one of the bona-fide sources was detected in three different map pairs (Figure 4); therefore, we found a total of seven bona-fide sources in rat brain tissue.*

Table S3. *QDM map pairs collected from rat brain samples. We classified map pairs as surface contamination based on large numbers of sources outside tissue (Figure S1). Sources in sample 4998_p5 were oriented in directions ~60° on average away from the expected direction (Figure S2). On the valid map pairs, binomial tests determined whether sources were randomly distributed in the FOV or resided in tissue.*

Sample	FOV	Polish	Classification	p_0	N_{tissue}	N_{epoxy}	p
4998_p5	1	1	Surface contamination	0.244			
4998_p5	1	2	Surface contamination	0.239			
4998_p5	1	3	Sources in wrong direction	0.246			
4998_p5	1	4	Sources in wrong direction	0.260			
4998_p5	1	5	Sources in wrong direction	0.230			
4998_p5	2	1	Sources in wrong direction	0.879			
4998_p5	2	2	Sources in wrong direction	0.882			
4998_p5	3	1	Surface contamination	0.408			
4999_p6	2	1	Surface contamination	0.399			
4999_p6	2	2	Valid map pair	0.356	4	0	0.016*
4999_p6	2	3	Valid map pair	0.366	1	0	0.366
4999_p6	2	4	Valid map pair	0.332	4	1	0.044*
5000_p13	1	1	Valid map pair	0.811	0	0	-
5002_p20	1	1	Valid map pair	0.303	0	0	-

p_0: hypothesized probability to find a particle given by the ratio of tissue area over FOV area; N_{tissue}: number of sources in tissue; N_{epoxy}: number of sources in epoxy; p: p-value of the binomial test (*, $p < 0.05$).

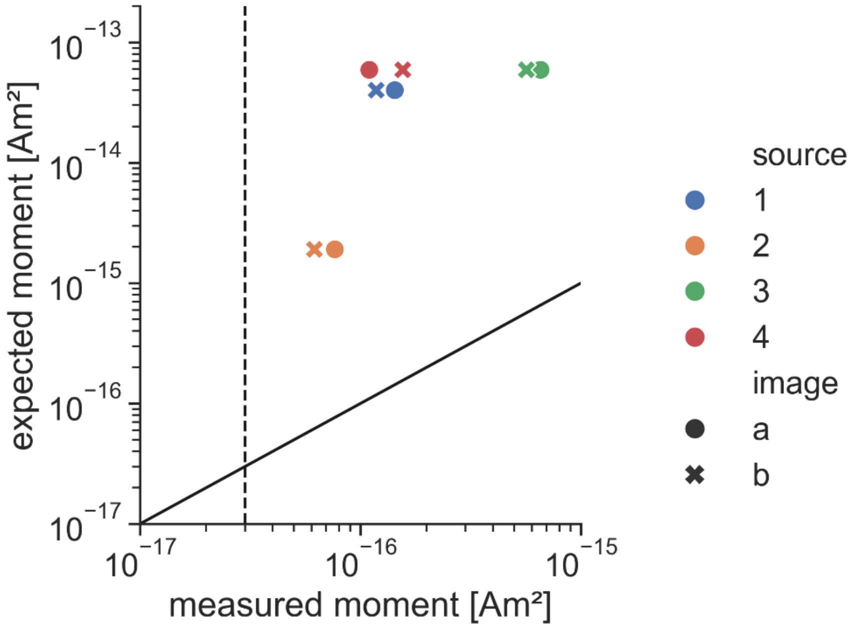

Fig. S3. *Magnetic moments of human brain samples exceeding the QDM noise threshold (3×10^{-17} Am^2, dashed line) plotted against the moment measured with the SQUID magnetometer scaled to the equivalent volume, assuming a QDM detection depth of 6 µm and a homogeneous distribution of magnetite in the tissue. The black line denotes a one-to-one relationship.*

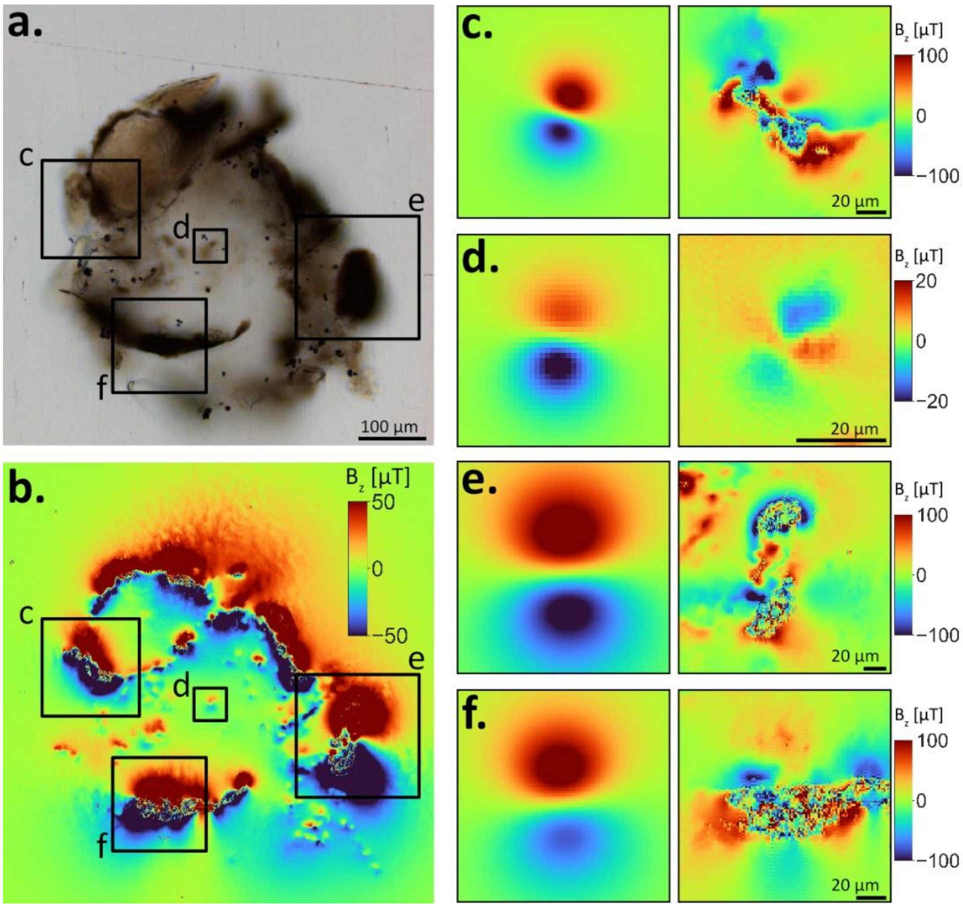

Fig. S4. *QDM image of a sample containing magnetotactic bacteria (MTB) as a control for the embedding process from LMU-Munich.* ***a****) Photomicrograph of the sample.* ***b****) Magnetic field map of the corresponding FOV. Saturated magnetic field map with non-dipolar signals due to the high signal strengths from the sample.* ***c-f****) Four areas were chosen to fit dipole models to the data (left panel) with corresponding residual magnetic field maps (right panel). Note that the color scales cover two to three orders of magnitude higher magnetic fields compared to maps of brain tissue. The complex, non-dipolar, and saturated magnetic field map resulted from the dense accumulation of magnetosomes. Embedding cells in MMA therefore did not alter intracellular magnetite nanoparticles.*

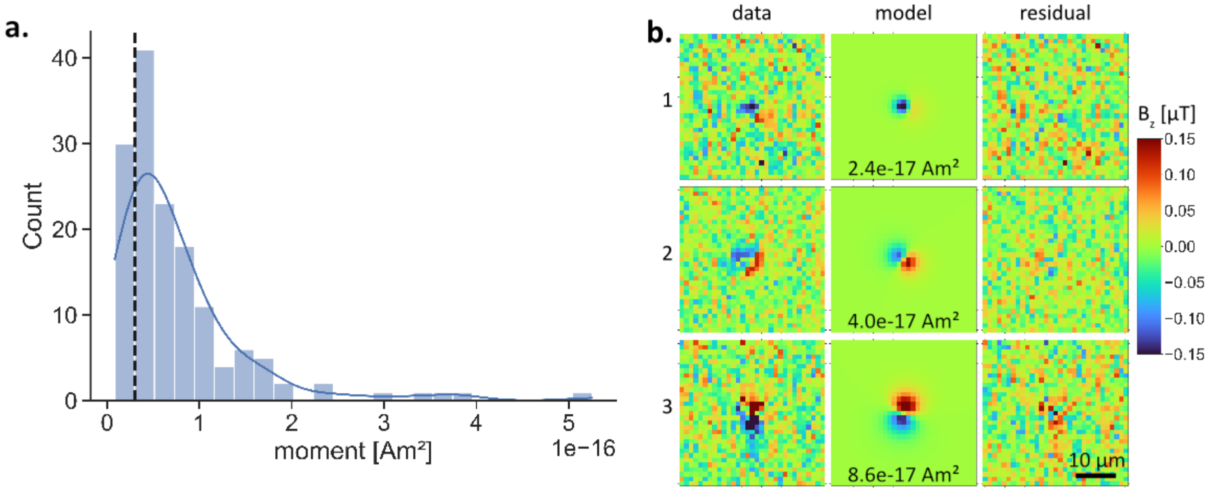

Fig. S5. *Sensitivity and noise level of the QDM in the experimental setup used for the present study.* ***a****) Histogram of magnetic moments from 147 of the smallest dipole signals found in eleven high-sensitivity QDM magnetic field maps. Counts peaked at a magnetic moment of* 4×10^{-17} *Am*^{2}*. Given that sources with this magnetic moment can reliably be identified in the high-sensitivity QDM maps, we consider* 3×10^{-17} *Am*^{2} *(black dashed line) as the smallest possible resolvable magnetic moment using our instrumental setup.* ***b****) Representative dipole signals used for the noise characterization. For three examples, the raw data, dipole model fit (with the magnetic moment), and the residuals after fitting are shown.*
